# Progress in Pulmonary Vein Stenosis: Lessons from Success in Treating Pulmonary Arterial Hypertension

**DOI:** 10.3390/children9060799

**Published:** 2022-05-29

**Authors:** Kathy J. Jenkins, Jeffrey R. Fineman

**Affiliations:** 1 Department of Cardiology, Boston Children’s Hospital, Boston, MA 02115, USA; kathy.jenkins@cardio.chboston.org; 2 Department of Pediatrics, University of California, San Francisco, CA 94143, USA

**Keywords:** pulmonary veins, pulmonary hypertension, vascular endothelium

## Abstract

Pulmonary vein stenosis (PVS) is a rare and poorly understood condition that can be classified as primary, acquired, status-post surgical repair of PVS, and/or associated with developmental lung disease. Immunohistochemical studies demonstrate that obstruction of the large (extrapulmonary) pulmonary veins is associated with the neointimal proliferation of myofibroblasts. This rare disorder is likely multifactorial with a spectrum of pathobiology. Treatments have been historically surgical, with an increasing repetitive interventional approach. Understanding the biology of these disorders is in its infancy; thus, medical management has lagged behind. Throughout medical history, an increased understanding of the underlying biology of a disorder has led to significant improvements in care and outcomes. One example is the treatment of pulmonary arterial hypertension (PAH). PAH shares several common themes with PVS. These include the spectrum of disease and biological alterations, such as vascular remodeling and vasoconstriction. Over the past two decades, an exponential increase in the understanding of the pathobiology of PAH has led to a dramatic increase in medical therapies that have changed the landscape of the disease. We believe that a similar approach to PVS can generate novel medical therapeutic targets that will markedly improve the outcome of these vulnerable patients.

## 1. Introduction

Pulmonary vein stenosis (PVS) is a rare and poorly understood condition that can be classified as primary, acquired, status-post surgical repair of PVS, and/or associated with developmental lung disease. Given its varied pathology and lack of national registries, prevalence and outcome data are inconsistent. Recent studies suggest a prevalence of ~1.7 cases per 100,000 children under 2 years old [1]. Survival or freedom from re-operation/stenosis 3–5 years after initial treatment is ~40–60% [1,2,3]. Thus, this rare but devastating disease is a significant source of morbidity and mortality in those affected.

Immunohistochemical studies demonstrate that obstruction of the large (extrapulmonary) pulmonary veins is associated with neointimal proliferation of myofibroblasts [4]. This can result in elevated pulmonary venous pressure, pulmonary edema, pulmonary arterial hypertension, and potentially cardiac failure and death. This rare disorder is likely multifactorial with a spectrum of pathobiology. Treatments have been historically surgical, with an increasing repetitive interventional approach [5]. Understanding the biology of these disorders is in its infancy; thus, medical management has lagged behind.

Throughout medical history, an increased understanding of the underlying biology of a disorder has led to significant improvements in the care and outcomes. Some of the more dramatic examples recently emerged from precision medicine approaches to many cancers. 

For example, the appreciation that mutations in the anaplastic lymphoma kinase (*ALK*) gene are associated with non-small-cell lung cancer led to the emergence of ALK1 inhibitors as an outstanding treatment strategy [6]. A pediatric disorder that was historically treated with surgery and/or interventional therapies where the care has been dramatically improved by medical management is infantile hemangiomas [7]. One report of an infant with a severe lesion who was serendipitously treated with propranolol for associated heart failure exhibited marked regression of the lesion following the initiation of therapy. This report led to what is now considered the gold standard therapy for these hemangiomas—beta blockers [8]. 

In contrast to most medical breakthroughs, in which a fundamental understanding of the biology leads to therapies, the medical treatment of infantile hemangiomas emerged by an astute observation, and the biological targets of beta blockers in these disorders remain incompletely understood. Potential mechanisms include vasoconstriction, decreased expression of *VEGF* and *bFGF* genes through the down-regulation of the RAF–mitogen-activated protein kinase pathway, and the triggering of apoptosis of capillary endothelial cells [9,10]. A more closely aligned pediatric pulmonary vascular disorder to pulmonary vein stenosis than hemangiomas is pulmonary arterial hypertension (PAH), a group of disorders whose treatment options and outcomes have vastly improved over the past two decades secondary to an increased understanding of the pathobiology [11].

## 2. Medical Treatment for Pulmonary Arterial Hypertension

PAH is a disease that is associated with a spectrum of disorders, such as congenital heart disease, infections, collagen vascular disease, and developmental lung diseases [12]. Thus, the underlying pathobiology is likely diverse and multifactorial. Despite these diversities, the underlying vascular remodeling, medial hypertrophy, abnormal extension of muscle to the periphery, intimal hyperplasia, and loss of small arterioles looks remarkably similar [13]. Although endothelial dysfunction has been well documented in many differing animal models of PAH and human disease, much of our understanding of early endothelial injury in the establishment and progression of PAH stems from studies related to congenital heart disease (CHD)-associated PAH [14]. 

Most forms of PAH present to medical attention with heart failure, late in the course of the disease. Thus, a window into the inciting event and early stages of the disease is lacking. CHD-associated PAH is unique in that we understand the PAH-inciting events; congenital cardiac defects resulting in increased pulmonary blood flow and pressure induce abnormal mechanical forces (shear stress and stretch) on the pulmonary vascular endothelium [15,16]. Importantly, regardless of the inciting event, vascular remodeling and PAH will induce similar pathologic mechanical forces suggesting that this pathobiology likely participates in the progression of many forms of PAH.

Beginning with landmark studies by Rabinovitch and colleagues who demonstrated structural endothelial cell abnormalities in children with CHD and increased pulmonary blood flow, and Celemajer and colleagues who demonstrated functional endothelial cell abnormalities in a similar group of patients, endothelial cell injury and dysfunction has been shown to be paramount to PAH [17,18,19]. These data include decreases in pulmonary vascular nitric oxide and prostacyclin production, as well as the increased production of endothelin-1 [14,20,21,22]. 

These changes promote an increase in vasoconstrictor activity and smooth muscle proliferation—two hallmarks of PAH. Within this pathobiological framework came the emergence of endothelial-based medical therapies that augment the nitric oxide and prostacyclin cascades and inhibit the endothelin cascade. Prior to 1995, before any currently available medical treatments for PAH, the average time from diagnosis to death for pediatric PAH was ~10 months [23]. With the emergence of these medical therapies, starting with IV epoprostenol in 1995 and expanding rapidly to our current approval of 17 medical therapies for adult PAH, more recent registry data demonstrates a 1-year survival of 96% and a 5-year survival of 74% for pediatric PAH [24]. 

In addition, as we learn more about the timing and combination of the endothelial-based drugs, we expect a continued improvement in patient outcomes [25]. As with certain cancers, the association of genetic aberrations and the development of PAH has become well established. In fact, current data suggest that ~40% of pediatric PAH has a demonstrated genetic aberration [26]. The identification of these genetic associations can aid in identifying an etiology and/or diagnosis, aid in identifying a mechanism of disease, aid in designing a treatment plan, and potentially identify novel therapeutic targets. 

The most common gene mutations involved in both adult and pediatric PAH are in BMPR2—a member of the TGF superfamily. These mutations account for ~80% of heritable PAH and 20% of sporadic PAH [27]. Similar to some cancers, an increasing understanding of the subsequent pathobiology of these patients has recently led to a novel therapeutic target. In fact, sotatercept, a novel fusion protein developed to normalize BMPR2 signaling has demonstrated promising results and represents the first non-endothelial-based PAH therapeutic target [28].

## 3. Medical Treatment for Pulmonary Vein Stenosis

Pulmonary vein stenosis holds many similarities to PAH. It likely represents a spectrum of disorders with differing underlying pathobiologies that, similar to PAH, include aberrations in shear stress, vasoconstriction, vascular remodeling, and inflammation [16,29]. In addition, the resulting increased pulmonary venous pressure from multiple vessel PVS may induce PAH by mechanisms that are poorly understood. 

Importantly, the potential genetic contribution to PVS is gaining appreciation; however, information is sparse. However, genetic syndromes occur in ~30% of cases [30]. The historic approach to PVS therapy has been surgical and, more recently, aggressive repetitive interventions. Medical therapies for these disorders are in their infancy, owing to the fundamental lack of understanding of the biology. This understanding has been further impaired by the lack of adequate animal models of the disease [31].

Human specimens have demonstrated neointimal proliferation of myofibroblasts—cells that exhibit dual myocyte and fibroblast differentiation—in the pulmonary veins of both normally connected veins and restenosed veins followed total anomalous repair [31,32]. A piglet model of PVS also demonstrated intimal hyperplasia of cells that underwent endothelial–mesenteric transformation [33]. Thus, potential sources of myofibroblasts include the proliferation of existing pericytes and fibroblasts in various vascular compartments [34]. 

These observations led to the evaluation of anti-proliferative medical therapies, such as vinblastine and methotrexate based on efficacy to treat desmoid tumors, an infant condition with a similar cellular basis [35]. Further human studies found immunoreactivity for the receptor tyrosine kinases, prompting the use of imatinib mesylate and bevacizumab to target inhibition of PDGFR and VEGFR [36,37]. The use of systemic sirolimus, an mTOR pathway inhibitor, is supported by a pig pulmonary vein obstruction study that demonstrated de-differentiation of smooth-muscle-cell-like cells in mTOR pathway activation [38]. 

Losartan, an angiotensin II type 1-receptor blocker, is also currently under study in patients with PVS. Utilizing a piglet model of banded pulmonary veins, Kato et al. demonstrated increased expression of transforming growth factor β (TGF-β) and Smad4 in the myofibroblasts of banded pulmonary veins [33]. Losartan attenuates TGF-β signaling, and losartan treatment demonstrated benefit in this piglet model and prompted its trial in humans [39]. 

It is noteworthy that many of these agents demonstrate significant adverse effects that include bone marrow and immunosuppression, gastrointestinal toxicity, and systemic hypotension [37,38,39,40]. Thus, results from these trials must be interpreted cautiously. Given the paucity of data in controlled trials, recommendations of a particular agent cannot be made at this time.

Similarities in the pathobiology between PVS and PAH, which include the effects of abnormal shear stress and vasoconstriction, have implicated the potential use of PAH medications for these disorders [16,29]. In addition to potentially attenuating pulmonary venous remodeling and vasoconstriction, these agents may theoretically attenuate the progression to PAH. 

For example, utilizing a swine pulmonary vein banding model, van Duin et al. demonstrated that the transition from post-capillary to pre-capillary pulmonary hypertension was associated with an upregulation of the edothelin-1 cascade, suggesting that endothelin-1 receptor antagonists may be a therapeutic target to attenuate this progression [41]. However, the potential therapeutic benefit of pulmonary vasodilators has not been rigorously investigated and should proceed with caution given the risk of pulmonary edema with multi-vessel disease. In fact, the use of pulmonary vasodilators in the treatment of PAH secondary to left-sided heart disease remains unproven and controversial [42].

Even with an improved understanding of the pathobiology of PVS, the evaluation of medical therapy will not be straightforward. In addition to the common challenges associated with designing studies for rare, heterogenous conditions, PVS has the added complexity of requiring surgical or catheterization-based therapy for patient survival. Indications and approaches to interventions are evolving, and differences in the timing and interventions themselves makes evaluating the efficacy of medical therapies even more difficult. Most patients undergo a variety of interventions prior to referral for medical treatment, and thus studies must consider the disease status at treatment onset in order to make legitimate comparisons. 

Study designs also need to consider the marked differences in disease aggressiveness between patients to be sure that comparisons do not include bias by intrinsic differences in underlying disease severity. Studies in referral populations are especially challenging, since it is difficult or impossible to understand how the studied population reflects the target population of interest given the variation in referral patterns as most patients with less aggressive disease are never referred.

## 4. Conclusions

In summary, the use of medical therapies for PVS is in its infancy but represents an exciting emerging focus. Current human studies are based upon limited human and animal biology and are preliminary in nature. In general, studies are poorly controlled with small sample sizes. In addition, the investigation of diverse patient populations is problematic; similar to PAH, PVS likely represents a spectrum of disease pathology. The expanding enthusiasm to understand the underlying pathobiology of PVS to develop targeted medical therapies is warranted and deserving of increasing focus and resources. 

However, patience and caution are also required. We must resist the temptation to adopt therapies based on anecdotal experience without rigorous investigation, particularly since the intrinsic disease risk for progression is quite variable, and importantly, many of these medications could have serious adverse effects. As with PAH, the emergence of multi-institutional collaborations will provide the framework needed to rigorously investigate new treatment regimens with robust study designs and ultimately improve the outcomes of these vulnerable patients.
